# Genotype and phenotype classification of 29 patients affected by Krabbe disease

**DOI:** 10.1002/jmd2.12007

**Published:** 2019-03-14

**Authors:** Anna M. H. Madsen, Flemming Wibrand, Allan M. Lund, Jakob Ek, Morten Dunø, Elsebet Østergaard

**Affiliations:** ^1^ Department of Clinical Genetics Copenhagen University Hospital Rigshospitalet Copenhagen Denmark

**Keywords:** *GALC* mutations, genotype‐phenotype analysis, globoid cell leukodystrophy, Krabbe disease

## Abstract

Krabbe disease is a rare neurodegenerative lysosomal storage disorder caused by mutations in the galactocerebrosidase gene, *GALC*. Krabbe disease usually affects infants, but has also been reported in older children and adults. Different phenotypes are described based on age at onset. The gene encoding the galactocerebrosidase enzyme was cloned and expressed in 1993, and up until today 117 mutations have been described. In a patient population of Northern European origin, a 30‐kb deletion and two missense mutations, c.1586C>T; p.T529M and c.1700A>C; p.Y567S, are expected to account for 50%‐60% of pathogenic alleles. In this study, we present information on genetic variation, enzyme activity, and phenotypes of 29 patients affected by Krabbe disease. Patient data were collected from patient files at the Department of Clinical Genetics, Rigshospitalet. Ten previously unreported mutations were identified, including four missense mutations; c.1142C>T; p.T381I, c.596G>T; p.R199M, c.443G>A; p.G148E, c.1858G>A; p.G620R, two nonsense mutations; c.863G>A; p.W288*, c.1214c>G; p.S405*, one splice site mutation; c.442+1G>A, one insertion; c.293insT and two deletions; c.1003_1004del, c.887delA. For all of the new mutations, we were able to classify them in phenotype groups. Furthermore, we present a combined allele frequency of the three frequent mutations p.T529M, p.Y567S, and the 30‐kb deletion of 62%, and we describe a broadening of the phenotypes associated with the mutations p.T529M and p.Y567S.

## 1 INTRODUCTION

Krabbe disease (OMIM #245200, globoid cell leukodystrophy, galactocerebrosidase deficiency) is an autosomal recessive neurodegenerative lysosomal storage disorder first described by the Danish neurologist Knud Haraldsen Krabbe in 1916.^1^ The disorder is caused by mutations in the galactocerebrosidase gene, *GALC*, leading to a low or total loss of activity of the encoded enzyme, galactocerebrosidase (GALC).^2^, ^3^ The deficiency results in an impaired degradation of galactocerebroside, a major myelin lipid, and of galactosylsphingosine, a cytotoxic compound.^2^, ^4^, ^5^ During myelin turnover, galactocerebroside is broken down to intermediary compounds that are reused in a remyelination pathway. Accumulation of galactocerebroside impairs remyelination and leads to the formation of multinucleated macrophages, globoid cells.^2^, ^4^, ^5^

Based on age at the onset of neurological symptoms, five different phenotypes of Krabbe disease have been described. Most patients manifest the early infantile form that has onset from birth until 6 months of age, and the most common symptoms are crying and irritability followed by fisting of hands, poor head control, poor feeding, stiffness, and loss of milestones.^6^ The late infantile form has onset when the child is between 7 and 12 months of age. The most common symptoms include loss of milestones, poor feeding, crying/irritability, and cortical fisting.^7^ The later onset group includes patients with onset at age 13 months to 10 years, and is characterized mainly by abnormal gait due to spastic paraparesis.^7^ The adolescent form with onset between age 11 and 20 years, and the adult form with onset past age 20 years, are rare phenotypes. Their main symptom is abnormal gait, and the disease may be nonprogressive for years.

The cDNA encoding the human ß‐galactocerebrosidase enzyme was cloned and expressed in 1993.^8^ *GALC* is localized on chromosome 14q31, it spreads over 58 kb and consists of 17 exons.^9^ The gene encodes an 80 kDa precursor protein, which is cleaved into a 50 and 30 kDa subunit.^8^, ^10^, ^11^ Until now, 237 mutations causing Krabbe disease have been cataloged in the Human Gene Mutation Database (professional subscription, August 2018).

Among the first mutations to be described was a 30‐kb deletion (c.1161+6532_ polyA+9kbdel).^12^, ^15^ This deletion starts near the middle of intron 10 and extends through to exon 17 plus an additional 9 kb. Consequently, it eliminates the coding region for the 30‐kDa subunit and 15% of the coding region for the 50‐kDa subunit.^15^ The 30‐kb deletion is invariably found to be associated with a c.550C>T transition on the same allele.^13^, ^14^, ^15^ The 30‐kb deletion is the most common mutation among patients with European ancestry.^16^ Two base substitutions, leading to the missense variants p.T529M and p.Y567S, are also frequently found in the European population, and together these three mutations are estimated to account for approximately 50%‐60% of pathogenic alleles in early infantile patients of European ancestry.^16^

In almost all individuals with Krabbe disease, galactocerebrosidase enzyme activity is reduced to between 0% and 5% of normal activity in leukocytes isolated from whole heparinized blood, or in cultured skin fibroblasts.^16^ Therefore, the diagnosis can be established by measurement of GALC enzyme activity in leukocytes or other tissue. The patient will also undergo molecular genetic analysis to identify both pathogenic alleles to aid in phenotype prediction,^16^ and for example, carrier analysis or prenatal diagnosis. Furthermore, low GALC activity can be caused by pseudodeficiency alleles, that is, *GALC* variants that reduce enzyme activity in vitro, but do not cause disease, which underscores the importance of molecular genetic analysis.

The only treatment of Krabbe disease available today is hematopoietic stem cell transplantation using bone marrow or cells from umbilical cord blood.^17^ The treatment is only effective when it is offered to presymptomatic infants.^17^

In 2006, the state of New York established a Krabbe disease screening program, with the purpose of identifying children at high risk of developing early infantile Krabbe disease, and to offer patients hematopoietic stem cell transplantation before onset of symptoms.^18^ The World‐Wide Krabbe Registry was established in connection to the screening program with the primary goal of determining whether clinical, biochemical, genetic, or neurodiagnostic parameters (including cerebral CT/MRI, cerebrospinal fluid analysis, electroencephalograms, flash visual evoked responses, brainstem auditory evoked responses, and nerve conduction velocities) could predict phenotype.^6^ Data from 122 patients with symptomatic Krabbe disease were available for the World‐Wide Krabbe Registry.^7^ However, neither biochemical, genetic, or neurodiagnostic studies could predict phenotype with certainty.^7^ Recently, the results from the first 8 years of the screening program were published.^19^ Of more than 1.9 million screened infants, five had enzyme activities and molecular genetic findings consistent with early infantile Krabbe disease and manifested clinical/neurodiagnostic abnormalities. Four infants underwent bone marrow transplantation, two patients died due to complication and the two surviving patients had severe developmental and motor delay.^19^, ^20^ However, earlier studies have shown that treated patients may maintain normal vision and hearing and normal cognitive development.^17^

This study presents data on genotype, enzyme activity, and phenotype of Krabbe patients diagnosed during a period of more than 35 years in Denmark. The nationwide cohort includes 29 patients, which makes it one of the largest mutation studies of *GALC* in a population of mainly Northern European ancestry. We aim at expanding the knowledge of the correlation between genotype and phenotype in patients with Krabbe disease, of use for already established and future screening programs for Krabbe disease.

## 2 MATERIALS AND METHODS

### 2.1 Patient data

This study includes 29 Krabbe patients who were diagnosed from 1979 to 2016 in Denmark. The patient cohort includes 25 unrelated patients and two families each comprising two patients. The Department of Clinical Genetics, Rigshospitalet is the only center in Denmark that performs diagnostics (biochemical and molecular genetics) of patients with Krabbe disease. The study is retrospective and comprises clinical data collected from patient files, biochemical and molecular genetic data. For the majority of the patients, information about the exact time of onset of disease was not available. However, for all patients except patient number 29, the time of the first contact to a pediatric ward at a local hospital was recorded. For the patients where information about age at onset of disease was available, we found that on average the first contact to a pediatric ward was 2 months following onset of signs. Thus, we have defined time of onset of disease as 2 months before the first contact to a pediatric ward. No clinical data were available for patient number 29, and this patient was left out of the classification of patients in phenotype groups and calculation of survival time. Patient number 13 was residing in Iceland and, therefore, we did not know the date of death, and she was left out of the calculation of survival.

Four patients were of non‐European origin (patients #8, 14, 25, 26). In the calculation of allele frequencies of the three known mutations: the 30‐kb deletion, c.1586C>T; p.T529M and c.1700A>C; p.Y567S these four patients were left out, to be able to compare our results to previously reported allele frequencies for the mutations in patients with European ancestry.

We did not have reliable measurements of GALC enzyme activity in leukocytes, and thus only enzyme activity results from cultured fibroblasts are reported.

### 2.2 Classification of phenotypes

When going through the literature of Krabbe disease phenotypes, we found that the literature regarding the later onset Krabbe phenotypes was confounded by differences in nosology. However, the definition of early infantile Krabbe disease as onset from birth till six full months of age is well accepted. For classification of the later onset group of phenotypes, we used the definition formulated by the World‐Wide Krabbe Registry.^6^ With a phenotypic study of 122 Krabbe patients, this definition is well supported.

### 2.3 *GALC* mutation nomenclature

*GALC* mutations have traditionally been described on the basis of their amino acid position in the mature enzyme, with p.M17 being designated as the first residue. Current HGVS (Human Genome Variation Society) nomenclature recommendations require proteins to be numbered from the first methionine of the complete 42‐residue signal sequence (NM_000153.3); this nomenclature is used throughout this article.

### 2.4 GALC sequencing

DNA was isolated from 6 mL EDTA blood samples using a standard desalting procedure. All coding exons of *GALC* including flanking intronic sequences were PCR amplified and sequenced directly using standard techniques. Identified mutations were confirmed in a new PCR and sequencing reaction. NM_000153.3 was used as reference sequence. The common 30 kb deletion was assessed by an in‐house developed three‐primed PCR‐based assay.

### 2.5 Measurement of galactocerebrosidase activity

Galactocerebrosidase activity was measured with an assay modified from Wiederschain et al.^21^ essentially as described in the “Laboratory protocol for enzyme analysis for Krabbe disease” provided by Moscerdam Substrates. Briefly, 5 μL of fibroblast homogenate (~5 μg protein) was mixed with 20 μL of substrate solution containing 0.4 mmol/L of 6‐hexadecanoylamino‐4‐methylumbelliferyl‐beta‐d‐galactoside, 5 mmol/L sodium taurocholate and 3 mmol/L oleic acid in 0.10 mol/L sodium phosphate/0.05 mol/L citrate buffer, pH 5.4. The reaction mixture was incubated at 37°C for 18 hours, and the reaction was stopped using 200 μL of 0.5 mol/L sodium carbonate buffer/0.25% Triton X‐100, pH 10.7. The fluorescence intensity of hexadecanoylamino‐4‐methylumbelliferone was measured in a Fluostar Optima microplate reader (BMG Labtech, Ortenberg, Germany) at excitation and emission wavelengths of 405 and 450 nm, respectively.

### 2.6 Measurement of chitotriosidase activity

Chitotriosidase activity was determined by incubating 5 μL of plasma with 100 μL 22 μmol/L 4‐methylumbelliferyl‐beta‐d‐N,N′,N″‐triacetylchitotriose in 0.20 mol/L sodium phosphate/0.10 mol/L citrate buffer, pH 5.2, for 1 hour at 37°C. The reaction was terminated by adding either 2 mL or 140 μL of 0.5 mol/L sodium carbonate buffer, pH 10.7, and the fluorescence of 4‐methylumbelliferone was read in a Perkin Elmer LS55 fluorometer or a Fluostar Optima microplate reader (BMG Labtech, Ortenberg, Germany) at excitation and emission wavelengths of 360 and 450 nm, respectively.

## 3 RESULTS

Bi‐allelic *GALC* mutations were identified in all 29 patients. Patients #3 and 22 were siblings, and patients #14 and 25 were first cousins and carried identical genotypes. Altogether, 24 patients were of Danish origin, one patient was of Icelandic origin (#13), and four patients were of non‐European origin (patients #8, 14, 25, and 26). One patient (#26) was born to consanguineous parents. Table 1 summarizes the 29 genotypes, along with phenotype data and enzyme activity.

**Table 1:** Clinical findings, and biochemical and genetic data of 29 Krabbe disease patients

Patient	Sex	Age of onset	Age at diagnosis	Age at death	Symptoms	Neuroimaging	GALC activity in fibroblasts (nmol/h/mg protein; reference 0.5‐2.1)	Chitotriosidase activity in plasma (nmol/h/mL; see footnotes for reference intervals)	Genetic variation, NM_000153.3		Traditional mutation description
									Allele 1	Allele 2	
*The early infantile form; (onset from birth till 6 mo of age)*											
1	F	0 mo	3 mo (enz)	3.5 mo	Irritability, poor feeding, hypertonia, seizures	N.a.	N.a.	N.a.	c.1161+6532_ polyA+9kbdel	c.1171_1175delCATTCinsA; p.H391IfsX65	IVS10del30kb/ ‐
2	M	1 mo	N.a.	8.5 mo	N.a.	N.a.	N.a.	N.a.	c.203C>T; p.S68F	c.887delA	S52F/‐
3	M	3 mo	7 mo (enz)	1 y 4 mo	N.a.	N.a.	0.01	N.a.	c.1586C>T; p.T529M	c.1586C>T; p.T529M	T513M/T513M
4	F	5.5 mo	7 mo (enz)	14 mo	Crying/irritability, hypertonia, cortical fisting, obstipation, visual loss, deafness, poor feeding, hypersensibilitet, hyper excitable	N.a.	0.02	N.a.	c.1161+6532_ polyA+9kbdel	c.1161+6532_polyA+9kbdel	VS10del30kb/VS10del30kb
5	M	1.5 mo	N.a.	2 y 8 mo	N.a.	N.a.	N.a.	N.a.	c.1586C>T; p.T529M	c.1700A>C; p.Y567S	T513M/Y551S
6	F	3 mo	6 mo (enz)	1 y	Hypertonia, seizures, swallowing and sucking problems, **↑**CSF protein	MRI: demyelination and brain stem atrophy	0.01	N.a.	c.1161+6532_ polyA+9kbdel	c.967G>A; p.G323R	IVS10del30kb/‐
7	M	4 mo	8 mo (enz)	1 y 5 mo	Swallowing problems, hypertonia, hyperhidrosis	MRI: degenerative changes	0.02	N.a.	c.1586C>T; p.T529M	c.1142C>T; p.T381I	T513M/‐
8	F	3 mo	6 mo (enz)	9 mo	Crying/irritability, psychomotor retardation myoclonus, lack of head control*,* abnormal contact	MRI: delayed myelination of basal ganglia and cerebelum	0.18	143^a^	c.860G>A; p.C287Y	c.596G>T; p.R199M	C271Y/‐
9	F	0 mo	5 mo (enz)	1 y	Crying/irritability, hypertonia, **↑**CSF protein	N.a.	0.02	30^a^	c.1161+6532_ polyA+9kbdel	c.293insT	IVS10del30kb/‐
10	F	0 mo	N.a.	7 mo	Poor feeding, myoclonus, hypertonia, **↑**CSF protein*	N.a.	0.02	368^a^	c.1161+6532_ polyA+9kbdel	c.1161+6532_ polyA+9kbdel	IVS10del30kb/IVS10del30kb
11	F	3.5 mo	7 mo (enz + seq)	1 y 2 mo	Megalencephaly psychomotor retardation, areflexia, failure to thrive, **↑**CSF protein^¤^, developmental delay	N.a.	N.a.	999^a^	c.1161+6532_ polyA+9kbdel	c.1214C>G; p.S405*	IVS10del30kb/‐
12	M	4.5 mo	7 mo (enz)	1 y 3 mo	Hypertonia, crying/irritability/macrocephaly	CT: atrophy	N.a.	475^a^	c.1161+6532_ polyA+9kbdel	c.1161+6532_ polyA+9kbdel	IVS10del30kb/IVS10del30kb
13	F	4 mo	6 mo (enz)	‐	Dystonia, crying/irritability	CT: white matter calcifications	N.a.	404^a^	c.1161+6532_ polyA+9kbdel	c.442+1G>A	IVS10del30kb/‐
14	F	1 mo	N.a.	1 y	N.a.	N.a.	0.02	N.a.	c.1858G>A; p.G620R	c.1858G>A; p.G620R	‐
15	F	0 mo	1 mo (enz + seq)	3 mo	Hypertonia, myoclonus, crying/irritability, hyperreflexia	MRI: cortex affected	N.a.	65^b^	c.1161+6532_ polyA+9kbdel	c.1161+6532 _polyA+9kbdel	IVS10del30kb/IVS10del30kb
16	M	4 mo	10 mo (enz)	2 y 4 mo	Psychomotor regression, hypotonia, passive	N.a.	N.a.	47^b^	c.1161+6532_polyA+9kbdel	c.443G>A; p.G148E	IVS10del30kb/‐
17	M	3 mo	N.a.	8 mo	N.a.	N.a.	0.02	N.a.	c.293insT	c.1003_1004del	‐
18	M	2.5 mo	N.a.	10.5 mo	N.a.	N.a.	0.02	N.a.	c.1161+6532_polyA+9kbdel	c.982C>T; p.Q328*	IVS10del30kb/Q312X
19	M	0 mo	N.a.	1 y 11 mo	N.a.	N.a.	0.02	N.a.	c.1161+6532_polyA+9kbdel	c.1161+6532_polyA+9kbdel	IVS10del30kb/IVS10del30kb
20	F	2 mo	15 mo (enz)	1 y 10 mo	N.a.	N.a.	N.a.	N.a.	c.1161+6532_ polyA+9kbdel	c.863G>A; p.W288*	IVS10del30kb/‐
21	F	6.5 mo	10 mo (seq)	2 y 7 mo	Psychomotor retardation	CT: central and cortical atrophy	N.a.	470^a^	c.1161+6532_polyA+9kbdel	c.1161+6532_polyA+9kbdel	IVS10del30kb/IVS10del30kb
*The late infantile form (onset from 7 till 12 of age)*											
22 (sister of patient 3)	F	10.5 mo	Postmortem	2 y 8 mo	N.a.	N.a.	N.a.	N.a.	c.1586C>T; p.T529M	c.1586C>T; p.T529M	T513M/T513M
23	M	10 mo	N.a.	2 y 7 mo	N.a.	N.a.	0.02	N.a.	c.1161+6532_polyA+9kbdel	c.1700A>C; p.Y567S	IVS10del30kb/ Y551S
24	M	7 mo	10 mo (enz)	3 y 9 mo	Psychomotor retardation, hypotonia, psychomotor regression, loss of acquired abilities, spasticity, macrocephaly	N.a.	0.06	66^a^ (age 7.5 mo) 144^a^ (age 10 mo)	c.1161+6532_polyA+9kbdel	c.1586C>T; p.T529M	IVS10del30kb/T513M
25 (first cousin of patient 14)	F	7 mo	N.a.	4 y 5 mo	N.a.	N.a.	0.02	N.a.	c.1858G>A; p.G620R	c.1858G>A; p.G620R	‐
26	M	11.5 mo	25 mo (enz)	2 y 11 m	Loss of motor function, spasticity	MRI: frontotemporal atrophy	0.27	350^a^ (age 17 mo) 655^a^ (age 1 y 9 mo)	c.1186C>T; p.R396W	c.1186C>T; p.R396W	‐
*The later onset group (onset from 13 mo till 10 y of age)*											
27	F	3 y	N.a.	Alive age 31 y	Spastic paraplegia, loss of gait*,* ataxia, dizziness, seizures, **↑**CSF protein level, abnormal EEG	MRI: demyelination	0.04	N.a.	c.1586C>T; p.T529M	c.1586C>T; p.T529M	T513M/T513M
28	M	13.5 mo	N.a.	2 y 7 mo	N.a.	N.a.	0.02	N.a.	c.1586C>T; p.T529M	c.1700A>C; p.Y567S	T513M/Y551S
*Unknown age at onset*											
29	M	N.a.	N.a.	1 y 9 mo	N.a.	N.a.	N.a.	N.a.	c.1586C>T; p.T529M	c.293insT	T513M/‐

Abbreviations: CSF, cerebrospinal fluid; enz, measurement of galactocerebrosidase activity in leukocytes; N.a., not available; seq, sequencing/PCR of *GALC*.

CSF protein: *4.0, ^¤^2.4 (ref < 0.4 g/L).

a Reference: 0‐110 nmol/h/mL.

b Reference 0‐42 nmol/h/mL.

### 3.1 Novel GALC gene mutations

We identified 10 previously unreported mutations in *GALC*, including four missense mutations; c.1142C>T; p.T381I, c.596G>T; p.R199M, c.443G>A; p.G148E, c.1858G>A; p.G620R, two nonsense mutations; c.863G>A; p.W288*, c.1214C>G; p.S405*, one splice site mutation; c.442+1G>A, one insertion; c.293insT and two deletions; c.1003_1004del and c.887delA. c.293insT were found in heterozygous state in three patients, p.G620R was found in homozygous state in two patients (first cousins), and the remaining mutations were found in heterozygous state only once.

In silico prediction of the pathogenicity of novel missense mutations was performed using SIFT (http://sift.jcvi.org/), Mutationtaster (http://www.mutationtaster.org/), and Polyphen2 (http://genetics.bwh.harvard.edu/pph2/), and the predictions indicated that all the novel missense mutations were expected to be damaging to the GALC protein (Table 2). The splice site mutation c.442+1G>A was analyzed by Alamut Visual Splicing module and the result indicated that this mutation damages the splice site, which could result in skipping of exon 5.

**Table 2:** In silico prediction of the effect of novel missense mutations

	SIFT score	Mutationtaster	Polyphen2
c.1142 C>T; p.T381I	0.01 damaging	Disease causing	0.999 probably damaging
c.596G>T; p.R199M	0.01 damaging	Disease causing	1.0 probably damaging
c.1858G>A; p.G620R	0.14 tolerated	Disease causing	0.962 probably damaging
c.443G>A; p.G148E	0.01 damaging	Disease causing	1.0 probably damaging

### 3.2 Frequency of previously reported *GALC* mutations in the Danish patient cohort

In this cohort of patients, the most frequent mutation was the 30‐kb deletion (c.1161+6532_polyA+9kbdel), comprising 44% (22/50) of pathogenic alleles among patients of Northern European origin. Six patients were homozygous for this deletion and 10 were heterozygous.

The second most frequent mutation in the cohort was the missense mutation p.T529M, comprising 20% (10/50) of pathogenic alleles. Three patients were homozygous for the substitution and four were heterozygous.

The third most frequently found mutations were the missense mutation p.Y567S and the previously unreported insertion c.293insT. Three patients were heterozygous for each of these mutations reaching an allele frequency of 6% (3/50) each.

A previously reported missense mutation p.R396W was found in homozygous state in one patient born to consanguineous parents of Turkish origin.

The remaining mutant alleles in this cohort were found in heterozygosity, and each of them was found only once. Among these, the nonsense mutation c.982C>T; p.Q328* has been reported twice before,^22^, ^23^ while the following mutations have each been reported once before; c.203C>T; p.S68F,^24^ c.860G>A; p.C287Y,^25^ c.1171_1175delCATTCinsA; p.H391Ifs*65,^26^ and c.967G>A; p.G323R.^26^

### 3.3 Galactocerebrosidase and chitotriosidase activity

GALC enzyme activity in fibroblasts was below the lower normal limit in all patients where measurements were available. Nine patients had chitotriosidase activity above the upper normal limit (patient #8, 10‐13, 15, 16, 21, and 26), one patient had chitotriosidase activity within the reference interval (patient #9), and one patient (patient #24) had one measurement of chitotriosidase in the reference interval (measured at age 7.5 months) and one measurement higher than the upper normal limit (measured at age 10 months).

### 3.4 Distribution of patients in groups of phenotypes and survival after onset of disease

In this cohort of Krabbe disease patients, 75% manifested the early infantile phenotype, 18% showed the late infantile phenotype, 7% had the later onset phenotype, and none of the patients manifested the adolescent or adult phenotypes.

**Figure 1:**
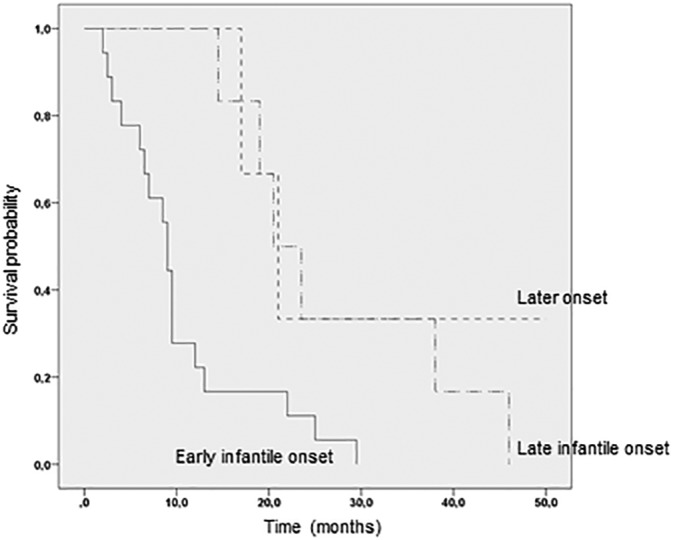
Kaplan‐Meier curve showing longer survival of Krabbe patients with the late infantile phenotype (n = 5) compared to patients with the early infantile form (n = 20). Patients with the later onset phenotype (n = 2) are shown for comparison

We estimated 1‐year, 5‐year, and 10‐year survivals for the three phenotype categories. For patients in the early infantile group, the survival was 40%, 0%, and 0%, respectively; for patients manifesting the late infantile phenotype, the survival was 100%, 0%, and 0%, respectively; and for patients manifesting the later onset phenotype survival was 100%, 50%, and 50%, respectively (Figure 1).

## 4 DISCUSSION

We here present the clinical, biochemical, and genotype data on 29 patients with Krabbe disease, including 10 previously unreported mutations. The new mutations include four missense mutations, two nonsense mutations, one splice site mutation, one insertion, and two deletions. The four missense mutations and the splice site mutation were predicted pathogenic in silico and found in patients that manifest the early infantile phenotype or, for the p.G620R in homozygous form, the late infantile phenotype. When performed, the pathogenicity of the novel missense mutations was supported by severely reduced GALC enzymatic activity.

The nonsense mutations; p.S405*, p.W288*, the insertion; c.293insT and the two deletions; c.1003_1004del and c.887delA are predicted loss of function mutations and thus, not expected to produce any functional GALC protein. In agreement, all of these mutations were found in patients manifesting the early infantile phenotype.

Among patients with Northern European ancestry in this cohort, we found a high allele frequency of three known mutations; the 30‐kb deletion and the missense mutations p.T529M and p.Y567S. Previously, the 30‐kb deletion has been reported to account for almost 40% of alleles in American patients with Northern European ancestry,^15^ 52% in unrelated Dutch patients,^14^ and 35% among patients from different European countries.^14^ Overall, the deletion is estimated to account for approximately 45% of pathogenic alleles in a population of European ancestry,^16^ and, accordingly, we found an allele frequency of 44%.

The p.T529M mutation has been reported to account for 5.3% of pathogenic alleles in unrelated Dutch patients^14^ and overall it is estimated to account for approximately 5%‐8% of pathogenic alleles in a population of European ancestry.^16^ Among patients of Northern European origin in our cohort, we found a 20% allele frequency of the p.T529M, which is much higher than that of both the Dutch population and the population with European ancestry. The p.Y567S mutation has been estimated to comprise 5%‐8% of alleles among people with European ancestry,^16^ which is in accordance with our results showing an allele frequency of 6%. In all, the two missense mutations comprised 26% of pathogenic alleles in this study, and together with the 30‐kb deletion, the three mutations comprised 62% of pathogenic alleles.

In our group of patients, we found no clear correlation between GALC enzyme activity and phenotype. In agreement, no such correlation has previously been observed.^6^, ^16^ Of note, though, patient #8 had a relatively high GALC activity and manifested the early infantile phenotype.

An elevated level of chitotriosidase activity is caused by activated macrophages and has previously been described in patients with Gaucher disease^27^ and other lysosomal storage diseases,^28^ however, the level in patients with Krabbe disease is not as high as in patients with Gaucher disease. Ten out of 11 Danish Krabbe disease patients had chitotriosidase activity higher than the upper normal level, while one patient with two loss of function mutations had a normal level of this enzyme. Similarly, an elevated level of this enzyme was previously found in 11 out of 15 patients with Krabbe disease.^29^ Although chitotriosidase enzyme activity appears to be an unspecific marker for Krabbe disease, it may still be useful in providing additional evidence of a storage process.

In the World‐Wide Krabbe Registry, 62% of patients manifested the early infantile phenotype, 10% showed the late infantile phenotype, 22% had the later onset phenotype, and 5% of the patients manifested the adolescent or adult phenotypes.^7^ In our cohort, a larger fraction manifested the early (75%) and late infantile (18%) onset phenotypes, compared to the World‐Wide Krabbe Registry and few or none manifested the later onset (7%), adolescent/adult (none) phenotypes. In a study of patients of Northern European ancestry, the early infantile group included 85%‐90% of the patients,^16^, ^30^, ^31^ which is higher than in our study. However, a larger part of our patients manifested the late infantile phenotype, and together the early and late infantile groups comprised 93% of Danish patients. Similar to our findings, Swedish Krabbe disease studies report no patients manifesting adolescent or adult phenotypes.^30^, ^32^ However, results from the New York State Krabbe disease screening program indicate that the later onset forms may be underdiagnosed.^19^, ^20^

In concordance with the results from the World‐Wide Krabbe Registry, we found longer survival in the groups of patients manifesting later onset and late infantile phenotypes, compared to the group of patients manifesting the early infantile phenotype. However, there are striking differences between the survival time after onset of symptoms among patients in the early infantile and late infantile onset groups in our cohort compared to the World‐Wide Krabbe Registry; for both phenotypes, the World‐Wide Krabbe Registry reports much higher 5 year (10% vs 0% for the early, and 50% vs 0% for the late infantile phenotype) and 10 year (4% vs 0% for the early and 50% vs 0% for the late infantile phenotype) survival rates than our study. Among our patients with the early infantile phenotype, the median age at death was 1.1 years of age, which is in agreement with findings in Swedish patients with an early infantile phenotype.^32^ Also, in our late infantile phenotype cohort, the median survival time following onset of disease was 2 years, which is comparable to a previous study of 50 patients with later onset phenotypes,^30^ where time from onset of disease until death was between 18 months and 4 years for the majority of children.^30^ The differences in survival time between ours and previously reported patients could be caused by national differences in active life support.

It has proven difficult to predict the phenotype of novel missense mutations or mutations found in a heterozygous state.^31^, ^33^ However, some mutations are considered severe and are expected to be associated with the early infantile phenotype when found in homozygosity, or compound heterozygous with another severe mutation, for example, the 30‐kb deletion or one of the two missense mutations p.T529M and p.Y567S.^6^, ^14^, ^15^, ^16^, ^26^, ^31^ In accordance with this, we identified all three mutations in the group of patients manifesting the early infantile phenotype. However, we also found both missense mutations associated with the late infantile phenotype (Table 1) as well as the later onset phenotype, including a patient homozygous for p.T529M who is currently alive at 29 years of age. In the literature, one patient homozygous for p.T529M had the late infantile phenotype and was still alive 52 months following onset of disease.^7^ So the p.T529M and p.Y567S mutations appear to be associated with both severe and milder phenotypes of Krabbe disease, suggesting the existence of additional disease modifying components.

The two families with two affected subjects showed intrafamilial phenotype variability with early infantile and late infantile phenotypes in both families (patients #3 and 22 are siblings, patients #14 and 25 are first cousins). Several previous papers have reported families with patients manifesting later onset phenotype in conjunction with either adolescent or adult onset phenotypes.^22^, ^30^, ^34^, ^35^, ^36^, ^37^ However, only three families have been reported previously to involve both early and later onset forms.^30^ The fact that different phenotypes are observed in related patients suggests that factors other than the *GALC* mutations alone influence the clinical course of Krabbe's disease, which makes prognosis prediction difficult.

In conclusion, we present the identification of 10 new *GALC* mutations. Furthermore, for each of them, we are able to associate the mutation to a phenotype. We report the highest allele frequency of the p.T529M mutation in one study, and together with the p.Y567S mutation, these two missense mutations comprise 26% of alleles in our patient population. This allowed us to study the clinical consequences of these alleles thoroughly. We found that the two missense mutations are associated with not only the early infantile phenotype as previously described, but also with the late infantile and later onset phenotypes. This is an important finding, because both we, and results from the World‐Wide Krabbe Registry, show that children manifesting either one of the later onset phenotypes have longer survival than patients with the early infantile phenotype. Inclusion of Krabbe disease in newborn screening programs may lead to presymptomatic hematopoietic stem cell transplantation, which is associated with a significant mortality and morbidity^18^, ^20^; thus, a thorough knowledge about phenotype and genotype correlations is of importance for existing and future screening programs.

## CONFLICTS OF INTEREST

All authors declare they have no competing interest.

## AUTHOR CONTRIBUTIONS

*Anna M. H. Madsen*: Conceived and designed the study, analyzed and interpreted the data; drafted the article and revised it critically for important intellectual content.

*Flemming Wibrand*: Conceived and designed the study, analyzed and interpreted the data; drafted the article and revised it critically for important intellectual content.

*Jakob Ek*: Conceived and designed the study, analyzed and interpreted the data; drafted the article and revised it critically for important intellectual content.

*Morten Dunø*: Conceived and designed the study, analyzed and interpreted the data; drafted the article and revised it critically for important intellectual content.

*Allan M. Lund*: Drafted the article and revised it critically for important intellectual content.

*Elsebet Østergaard*: Conceived and designed the study, analyzed and interpreted the data; drafted the article and revised it critically for important intellectual content.

## ETHICS APPROVAL AND PATIENT CONSENT

The study was approved by the Danish Patients Safety Authority (protocol number: 3‐3013‐2462/1) and the Capital Region of Denmark (protocol Rh‐2018‐20‐6158). This was a retrospective study and according to the Danish Patients Safety Authority patient consent is not necessary in these cases.

## DATA AND MATERIAL AVAILABILITY

All data generated or analysed during this study are included in this published article.
